# The impact of long-term PM_2.5_ exposure on specific causes of death: exposure-response curves and effect modification among 53 million U.S. Medicare beneficiaries

**DOI:** 10.1186/s12940-020-00575-0

**Published:** 2020-02-17

**Authors:** Bingyu Wang, Ki-Do Eum, Fatemeh Kazemiparkouhi, Cheng Li, Justin Manjourides, Virgil Pavlu, Helen Suh

**Affiliations:** 1 Khoury College of Computer Sciences, Northeastern University, 440 Huntington Ave, Boston, MA 02115 USA; 2 Department of Civil & Environmental Engineering, Tufts University, Medford, MA USA; 3 Bouvè College of Health Sciences, Northeastern University, Boston, MA USA

**Keywords:** Air pollution, Chronic exposure, PM_2.5_, Environmental epidemiology, Cardiovascular disease mortality, Respiratory disease mortality, Cancer mortality

## Abstract

**Background:**

The shape of the exposure-response curve for long-term ambient fine particulate (PM_2.5_) exposure and cause-specific mortality is poorly understood, especially for rural populations and underrepresented minorities.

**Methods:**

We used hybrid machine learning and Cox proportional hazard models to assess the association of long-term PM_2.5_ exposures on specific causes of death for 53 million U.S. Medicare beneficiaries (aged ≥65) from 2000 to 2008. Models included strata for age, sex, race, and ZIP code and controlled for neighborhood socio-economic status (SES) in our main analyses, with approximately 4 billion person-months of follow-up, and additionally for warm season average of 1-h daily maximum ozone exposures in a sensitivity analysis. The impact of non-traffic PM_2.5_ on mortality was examined using two stage models of PM_2.5_ and nitrogen dioxide (NO_2_).

**Results:**

A 10 *μg* /m^3^ increase in 12-month average PM_2.5_ prior to death was associated with a 5% increase in all-cause mortality, as well as an 8.8, 5.6, and 2.5% increase in all cardiovascular disease (CVD)-, all respiratory-, and all cancer deaths, respectively, in age, gender, race, ZIP code, and SES-adjusted models. PM_2.5_ exposures, however, were not associated with lung cancer mortality. Results were not sensitive to control for ozone exposures. PM_2.5_-mortality associations for CVD- and respiratory-related causes were positive and significant for beneficiaries irrespective of their sex, race, age, SES and urbanicity, with no evidence of a lower threshold for response or of lower Risk Ratios (RRs) at low PM_2.5_ levels. Associations between PM_2.5_ and CVD and respiratory mortality were linear and were higher for younger, Black and urban beneficiaries, but were largely similar by SES. Risks associated with non-traffic PM_2.5_ were lower than that for all PM_2.5_ and were null for respiratory and lung cancer-related deaths.

**Conclusions:**

PM_2.5_ was associated with mortality from CVD, respiratory, and all cancer, but not lung cancer. PM_2.5_-associated risks of CVD and respiratory mortality were similar across PM_2.5_ levels, with no evidence of a threshold. Blacks, urban, and younger beneficiaries were most vulnerable to the long-term impacts of PM_2.5_ on mortality.

## Background

Studies have demonstrated associations between long-term exposure to airborne fine particles (PM_2.5_, particulate matter with an aerodynamic diameter ≤ 2.5 *μm*) and increased all-cause [1–7] and cardiovascular (CVD) and to a lesser extent respiratory and lung cancer mortality [3, 4, 8]. In our paper of almost 19 million Medicare beneficiaries [4], for example, we showed 12 month average PM_2.5_ prior to death to be associated with increased all-cause, cardiovascular, respiratory, and lung cancer mortality, including from specific causes such as chronic obstructive pulmonary disease (COPD) and pneumonia mortality. While our and other studies provide key evidence of PM_2.5_’s impacts on mortality from several specific causes of death, their findings were based on largely white and higher socio-economic status (SES) cohorts from specific locations [2, 9, 10] or living in urban areas close to air pollution monitoring sites [4]. More recently, studies have expanded their geographic scope through the use of spatio-temporal models to predict exposures for participants living away from air pollution monitoring sites [5, 11, 12], although most still largely focus on white, urban and higher SES populations [11, 12] or on all-cause [5] or CVD-related [13, 14] causes of death. Of note, one study of California elderly found higher risks for CVD mortality for rural as compared to urban populations [15]. While this study controlled for key demographic factors, including race, SES, and education level, the contribution of these factors to the higher mortality risks experienced by rural populations was not examined [15]. Correspondingly, a study of adult participants in the National Health Interview Survey found elevated PM_2.5_-associated CVD mortality risks, with no difference by race, possibly the result of the broad age range of their cohort, their relatively small sample size, and their time invariant PM_2.5_ exposure estimates [13]. New approaches are needed to examine PM_2.5_-associated risks of mortality more comprehensively, especially with regards to differentiation of risks for specific causes of death, for potentially susceptible sub-populations, and at low PM_2.5_ exposures.

The advent of machine learning methods allows us to comprehensively examine PM_2.5_ impacts on mortality in “big” populations as provided by administrative data, allowing examination of the impact of low PM_2.5_ exposures understudied groups, and less common causes of deaths. In this paper, we describe associations between long-term PM_2.5_ and mortality from specific causes in all Medicare beneficiaries living in the conterminous US from 2000 through 2008. We examined the shape of the exposure-response curve for PM_2.5_ and the impact of low PM_2.5_ exposures for specific causes of death for the entire population and for specific subgroups. We further examined the impact of non-traffic PM_2.5_ on mortality and of confounding of these associations by SES and confounding and effect modification by sex, race, age, SES, and urbanicity.

## Methods

Our study was approved by the Institutional Review Board of Tufts and Northeastern Universities.

### Medicare mortality dataset

We compiled enrollment data from the Centers for Medicare and Medicaid Services for 53 million Medicare beneficiaries (65–120 years) living in the US between 2000 and 2008. Using the International Classification of Disease (ICD-10) codes from the National Death Index, we extracted mortality from non-accidental and accidental causes, CVD, respiratory, cancer, ischemic heart (IHD) and cerebrovascular (CBV) disease, congestive heart failure (CHF), chronic obstructive pulmonary disease (COPD), pneumonia, and lung cancer.

### Pollution exposure

Daily PM_2.5_ estimates on a 6-km grid were obtained from validated spatio-temporal generalized additive mixed models [14]. Model inputs included PM_2.5_ data from the U.S. Environmental Protection Agency (EPA), meteorological and geospatial covariates, and traffic-related PM estimated using a Gaussian line-source dispersion model. The daily PM_2.5_ model performed well, with a cross-validation R^2^ of 0.76, with low bias and high precision [14]. We obtained monthly NO_2_ concentrations estimated on a 100 m grid from [16]. The model performed well, explaining 82 and 76% of the spatial and temporal variability, respectively, with low bias (21%) and error (2.4 parts per billion, ppb). For both pollutants, we matched beneficiaries to the grid point closest to their ZIP code centroid, accounting for residential moves, and averaged estimated values to obtain the 12-month average prior to death, hereafter referred to as “12-month average exposure”. We also estimated warm season average 1-h maximum ozone exposures for a subset of beneficiaries living in ZIP codes within 6 miles of air quality monitors from the US EPA (Environmental Protection Agency) AQS (Air Quality System) for 2000 through 2008, following methods described in [17].

### Urbanicity and SES

For each ZIP code, we assessed urbanicity (urban vs. non-urban) using Categorization B from the Rural Health Research Center (RHRC) [18] and SES using the annual mean gross adjusted income from the US Internal Revenue Service (IRS) Statistics of Income Division database. [SES for the missing 2000 and 2003 years was estimated as the 2001 and 2004 values, respectively.] To examine effect modification, we classified ZIP codes as “high”, “medium”, or “low” based on tertiles of the distribution of adjusted incomes across ZIP codes.

### Analytical models

We examined the association of 12-month average PM_2.5_ exposures and cause-specific mortality using Cox proportional hazards (Cox PH) models with strata for age, race, sex, and ZIP code in base models and additionally controlling for ZIP code and state SES in adjusted models. To take full advantage of the monthly resolution of our mortality data, we fit each of our models using 3.8 billion person-months of follow-up and analyzing all data simultaneously. We categorized age into 1-year intervals, with 90+ years included as 1 age interval to avoid excessive zero counts. For each cause of death, we examined effect modification using interaction terms for age, sex, race, and ZIP code-level urbanicity in SES-adjusted models and SES in base models. As a sensitivity analysis, we also fit base and SES-adjusted models additionally controlling for the warm-season average of daily 1-h maximum ozone exposures, with this analysis based on a subset of 22+ million Medicare beneficiaries who lived in ZIP codes within 6 miles of an ozone monitoring station. All results are expressed as the risk ratio (RR) per 10 *μg* /m^3^ increase in 12-month average PM_2.5_.

To characterize non-linearities in the PM_2.5_-mortality association, we fit SES-adjusted models using restricted cubic splines (RCS) with three knots [19] for all examined causes of death. RCS models with three knots were chosen as our main model, given its superior performance to models with 4 and 5 knots (Figure S4). To further examine effects of low PM_2.5_ exposures, we fit SES-adjusted models restricted to beneficiaries living in ZIP codes with average PM_2.5_ concentrations below 8, 10 or 12 *μg* /m^3^. In sensitivity analyses, we fit SES- and ozone-adjusted non-linear models for a subset of 22 million Medicare beneficiaries living near EPA ozone monitors.

While we did not control for potential confounding by NO_2_ due to strong correlations between PM_2.5_ and NO_2_ (*r* = 0.59), we used NO_2_ exposures to estimate the impact of non-traffic PM_2.5_ exposures on cause-specific mortality. We did so in two-stages, regressing 12-month PM_2.5_ on NO_2_ and using its residuals as the exposure measure in health models. Since NO_2_ originates primarily from traffic-related sources [20, 21], RRs from the second stage models can be interpreted as the mortality risk from PM_2.5_ that is unrelated to traffic sources. Note that our approach allows us to compare RRs associated with non-traffic PM_2.5_ to those for total PM_2.5_ but not to traffic-related PM_2.5_. To assess the validity of our approach, we compared 1-year average residuals of PM_2.5_ on NO_2_ to 1-year average elemental carbon (EC), sulfate, and sulfur concentrations measured at US EPA chemical speciation network (CSN) sites by ZIP code and year. Comparisons with EC were limited to those sites that analyzed EC using the total optical transmittance method, since these sites were located predominantly in urban and suburban areas, where the majority of our beneficiaries lived.

Since conventional statistical packages such as R and SAS are not able to analyze our large-scale data due to memory and processing limitations, we implemented both linear and non-linear Cox PH methods in Java. Our implementation overcame memory and processing limitations of conventional software packages, using data grouping and linkage methods, optimization techniques, and multi-threading, fitting our models for 53 million beneficiaries and 3.8 billion person-months of follow-up simultaneously in approximately 10 minutes. Our implementation of Cox PH is described in Appendix S1 and is hosted on GitHub (https://github.com/Rainicy/survival).

## Results

Our study population includes approximately 53 million Medicare enrollees living in over 41,000 US ZIP codes between 2000 and 2008, with approximately 3.8 billion enrollee-months of follow-up (Table 1). During the study period, approximately 16 million deaths were reported, with 15 million deaths from non-accidental causes, 6.4 million from CVD, 1.8 million from respiratory disease, and 3.6 million from cancer. More than 50% of CVD mortality was from IHD, 18% from CBV, and 7.4% from CHF. More than 50% of respiratory deaths were attributed to COPD and 26% to pneumonia; 28% of cancer deaths were from lung cancer. Seventy-four percent of beneficiaries lived in urban areas, with beneficiaries living in low income ZIP codes residing primarily in non-urban areas (Table S2). The overall mean 12-month PM_2.5_ concentration was 10.32 *μg* /m^3^ (sd = 3.15), with mean concentrations higher in urban as compared to non-urban ZIP codes. Correlations between 12-month PM_2.5_ and NO_2_ and O_3_ equaled 0.59 and 0.24, respectively. Note that correlations between PM_2.5_ and ozone were stronger when analyzed by region, equaling 0.31 in the Midwest, 0.23 in the West, 0.43 in the Northeast, and 0.46 in the South.

**Table 1:** Characteristics Medicare enrollees aged 65–120 Years, United States, from 2000 to 2008

Characteristic	ICD-10	Number	%
No. of enrollees		52,954,845	
No. of ZIP Code		41,630	
No. of Person-Month		3,743,349,849	
No. of deaths			
All Causes		15,843,982	100.0^a^
Non-accidental	A-R	15,324,059	96.7
Accidental	V-Y	381,685	2.4
All Cardiovascular (CVD)	I00-I99	6,371,713	40.2
Ischemic heart disease (IHD)	I20-I25	3,323,527	21.0
Cerebrovascular disease (CBV)	I60-I69	1,147,050	7.2
Congestive heart failure (CHF)	I50	471,127	3.0
All Respiratory	J00-J99	1,777,076	11.2
COPD	J40-J44	944,665	6.0
Pneumonia	J12-J18	462,736	2.9
All Cancer	C-D	3,576,207	22.6
Lung cancer	C34	988,643	6.2

*Abbreviations*: *COPD* Chronic Obstructive Pulmonary Disease

^a^ The numbers or the percentages from sub-causes of death do not sum to all causes of deaths or 100%, because additional other causes of death are not reported in this table

### PM_2.5_ and cause-specific mortality

Table 2 shows RRs associated with 12-month PM_2.5_ for mortality by cause in base and SES-adjusted models, with RRs additionally adjusting for warm-season average of daily 1-h maximum ozone presented in Table S3. In base models, RRs were positive and statistically significant for all causes of death, except for accidental mortality. RRs were attenuated after adjustment for SES, with RRs for CHF and lung cancer no longer significant. In SES-adjusted models, PM_2.5_-associated RRs were highest for CVD-related causes (1.088; 95% CI (Confidence Interval): 1.078, 1.098), including IHD (1.126; 95% CI: 1.112, 1.140) and CBV (1.126; CI: 1.103, 1.150). Although lower, PM_2.5_-associated risks for mortality from respiratory-related and cancer (1.025; 95% CI:1.012, 1.038) mortality were significant and positive. RRs in base and SES-adjusted models were essentially unchanged after additional adjustment for ozone (Table S3).

**Table 2:** Mortality risk ratios (95% CI) associated with a 10 *μg*/*m*^3^ increase in 12-month average PM_2.5_: Base and SES-Adjusted models, by cause of death, US 2000–2008

Causes of Death	PM_2.5_		Non-Traffic^a^ PM_2.5_	
	Base Model^b^	SES-Adjusted^c^	Base Model^d^	SES-Adjusted^e^
All Causes	1.235 (1.229,1.242)	1.050 (1.044,1.056)	1.075 (1.067,1.082)	1.015 (1.008,1.022)
Non-accidental	1.244 (1.238,1.251)	1.051 (1.045,1.057)	1.070 (1.062,1.077)	1.014 (1.007,1.021)
Accidental	0.922 (0.891,0.955)	0.998 (0.960,1.037)	1.001 (0.956,1.047)	1.018 (0.972,1.066)
All Cardiovascular	1.683 (1.669,1.696)	1.088 (1.078,1.098)	1.178 (1.165,1.191)	1.016 (1.005,1.028)
IHD	1.975 (1.954,1.996)	1.126 (1.112,1.140)	1.258 (1.239,1.277)	1.027 (1.011,1.043)
CBV	1.955 (1.919,1.992)	1.126 (1.103,1.150)	1.270 (1.238,1.303)	1.057 (1.029,1.085)
CHF	1.116 (1.082,1.150)	0.986 (0.953,1.021)	1.007 (0.966,1.050)	0.970 (0.930,1.012)
All Respiratory	1.241 (1.223,1.260)	1.056 (1.038,1.074)	1.048 (1.026,1.070)	0.989 (0.968,1.010)
COPD	1.045 (1.023,1.067)	1.023 (0.999,1.047)	1.005 (0.977,1.033)	0.998 (0.969,1.027)
Pneumonia	1.769 (1.721,1.819)	1.078 (1.044,1.114)	1.184 (1.138,1.233)	0.943 (0.905,0.982)
All Cancer	1.160 (1.147,1.172)	1.025 (1.012,1.038)	1.054 (1.038,1.069)	1.016 (1.001,1.031)
Lung cancer	1.078 (1.056,1.100)	0.995 (0.972,1.018)	1.009 (0.981,1.038)	0.986 (0.958,1.014)

*Abbreviations*: *IHD* ischemic heart disease, *CBV* cerebrovascular disease, *CHF* congestive heart failure, *COPD* chronic obstructive pulmonary disease, *SES* socio-economic status, *PM*_*2.5*_ fine particle, aerodynamic diameter < 2.5 *μm*

^a^ Non-traffic PM_2.5_ estimated as the residual of PM_2.5_ regressed on NO_2_ concentrations for each ZIP code

^b^ Estimated using Cox PH models with strata for age (1 year age categories with 90+ year old as one category), race (white, non-white), sex (male, female) and ZIP Code (41,630 ZIP codes). Results are expressed as risk ratio and 95% CIs per 10 *μg*/*m*^3^ increase in 12-month average PM_2.5_

^c^ Estimated using Cox PH models with strata for age, race, sex, ZIP code, SES

^d^ Two-stage analysis: (1) regression of 12-month PM_2.5_ on NO_2_ and (2) residuals of stage 1 (as non-traffic PM_2.5_) as the exposure measure in Cox PH model with strata for age, race, sex, ZIP code

^e^ Two-stage analysis: (1) regression of 12-month PM_2.5_ on NO_2_ and (2) residuals of stage 1 (as non-traffic PM_2.5_) as the exposure measure in Cox PH model with strata for age, race, sex, ZIP code, SES

**Fig. 1:**
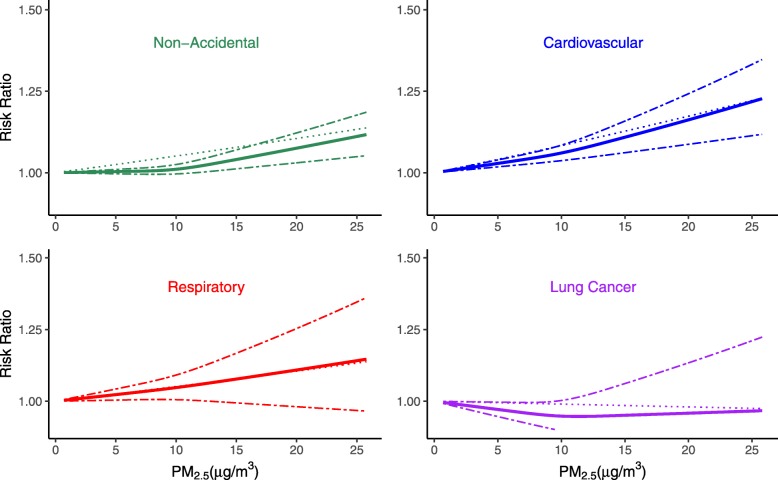
Non-linear and Linear Association of PM_2.5_ and Non-Accidental, Cardiovascular, Respiratory and Lung Cancer Mortality, SES-Adjusted. The non-linear and linear association of PM_2.5_ on cause-specific mortality: Non-Accidental, Cardiovascular, Respiratory and Lung Cancer. All analyses are conducted by Cox Proportional Hazard model with strata for age, sex, race, ZIP code and adjusted for SES. Bold solid line represents non-linear association estimated using restricted cubic spline (3 knots); dashed line represents 95% CIs for the non-linear, and dotted line represents linear association. RRs are based on comparisons to RRs for 0 *μg*/*m*^3^_._ Abbreviations: SES (socio-economic status), PM_2.5_ (fine particle, aerodynamic diameters < 2.5 *μm*)

The shape of the exposure-response functions for specific causes of death were examined in SES-adjusted models (Fig. 1, Figure S1, Table S5). We found RRs to increase monotonically with increased PM_2.5_ for all CVD and respiratory causes of death, except for CHF and pneumonia. For both CHF and pneumonia mortality, RRs were higher for PM_2.5_ exposures below as compared to above approximately 10 *μg* /m^3^. RRs were statistically significant and positive across the range of PM_2.5_ exposures for pneumonia, but were no longer significant for CHF at higher PM_2.5_ exposures (Figure S1). For non-accidental mortality, we found significant and positive RRs across the range of PM_2.5_ exposures, with no evidence of a threshold. The shape of the exposure-response curve, however, was sub-linear, with lower RRs when PM_2.5_ exposures were lower than 10 *μg* /m^3^ (Fig. 1). These findings are largely consistent with those from our analyses restricted to beneficiaries living in ZIP codes with PM_2.5_ concentrations below 8, 10, or 12 *μg* /m^3^ (Table S4). Sensitivity analyses showed control for ozone had no effect on the shape of the exposure-response curves for non-accidental and CVD mortality (Figure S2). However, the exposure-response curve for respiratory mortality became supra-linear after adjustment for ozone, with higher RRs when PM_2.5_ exposures were below as compared to above 10 *μg* /m^3^ for respiratory mortality (Figure S2).

**Fig. 2:**
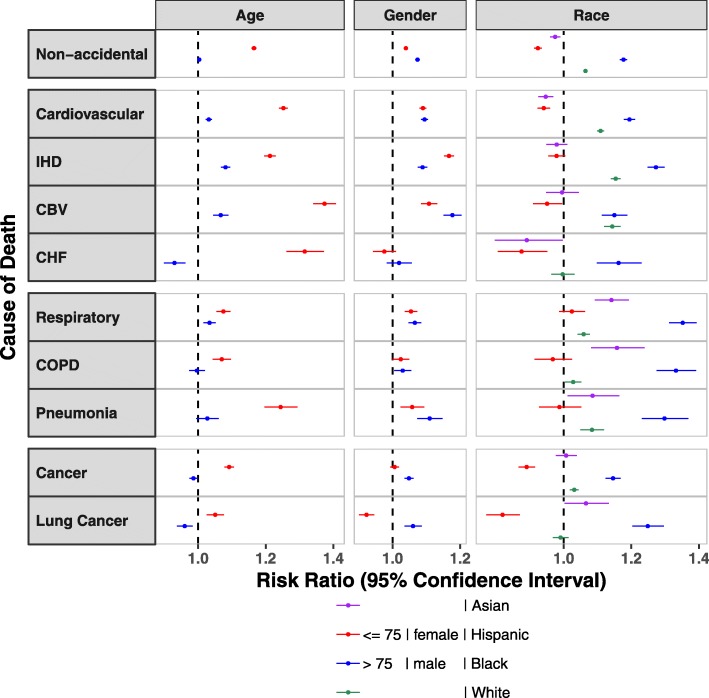
Modification of the SES-adjusted Association between PM_2.5_ and Cause-specific Mortality by Age, Sex, and Race. For each cause of death, we examined effect modification using interaction terms for age, sex and race respectively in the SES-adjusted models. Results are expressed as the risk ratio and 95% CIs per 10 *μg*/*m*^3^ increase in 12-month average PM_2.5_. Abbreviations: IHD (Ischemic heart disease), CBV (Cerebrovascular disease), CHF (Congestive heart failure), COPD (Chronic Obstructive Pulmonary disease), SES (Socio-Economic Status), PM_2.5_ (particles with aerodynamic diameters < 2.5 *μm*). Note: Each subgroup in the death-group box follows the same order defined in the figure legend

In SES-adjusted models, we found PM_2.5_-associated risks of death to vary by beneficiary characteristics (Fig. 2, Table S5). PM_2.5_-associated mortality risks differed most by race, with Black beneficiaries having the highest PM_2.5_-associated RRs for all causes of death, except for CBV, for which risks were similar for Blacks and Whites. For Whites, mortality risks were significant and positive for all causes of death, with lower risks than Blacks for non-accidental and CVD mortality and both Blacks and Asians for respiratory and lung cancer mortality. Risks of respiratory and lung cancer mortality for Asians were positive and second only to Blacks for respiratory and lung cancer mortality, but were null or negative for non-accidental and CVD mortality. PM_2.5_-mortality risks for Hispanics were protective or null for all causes of death. By age, PM_2.5_-associated RRs were higher for younger (≤75 years) as compared to older beneficiaries for all causes of death. While lower, RRs for older beneficiaries were positive and statistically significant for CVD-related (except for CHF), all respiratory, and pneumonia mortality. Differences in PM_2.5_-associated risks of mortality by sex were small, with significant positive risks for both men and women for all causes of death, except for CHF and lung cancer, with null risks for both sexes and for women, respectively (Figure S3). The shape of the exposure-response curves when stratified by age, sex, and race largely mirrored that for the entire population, with linear RRs for CVD and respiratory mortality and lower RRs at low as compared to high PM_2.5_ levels for non-accidental and cancer mortality, irrespective of age, sex, and race (Figure S3a-c).

**Fig. 3:**
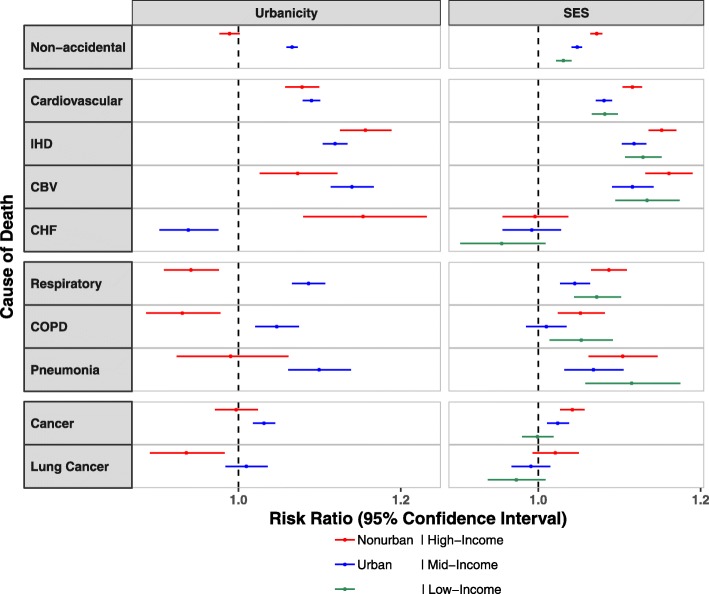
Modification of the SES-adjusted Association between PM_2.5_ and Cause-specific Mortality by Urbanicity and SES. For each cause of death, we examined effect modification using interaction terms for urbanicity and SES respectively in the SES-adjusted models. Results are expressed as the risk ratio and 95% CIs per 10 *μg*/*m*^3^ increase in 12-month average PM_2.5_. Abbreviations: IHD (ischemic heart disease), CBV (cerebrovascular disease), CHF (congestive heart failure), COPD (chronic obstructive pulmonary disease), SES (socio-economic status), PM_2.5_ (fine particles, aerodynamic diameters < 2.5 *μm*). Note: Subgroups follow the same order as in figure legend

We showed neighborhood characteristics to modify associations of PM_2.5_ and mortality (Fig. 3, Table S5). Beneficiaries living in urban as compared to non-urban ZIP codes had higher PM_2.5_-associated mortality risks for non-accidental, respiratory, and cancer mortality, with similar risks for CVD-related mortality. RRs for beneficiaries living in non-urban areas were positive and statistically significant for CVD-related causes of death, but were null for non-accidental, respiratory-related, cancer and lung cancer mortality. The shape of the exposure-response curves for urban areas and for beneficiaries living near monitoring sites were similar to that for all ZIP codes (Figure S3, S5). For SES, we found generally similar PM_2.5_-associated risks for all causes of death when all data were examined; however, when limited to urban areas, we found higher risks of increased CVD- and respiratory-related mortality for beneficiaries living in low as compared to high SES ZIP codes. The shapes of the exposure-response curves for ZIP codes with high and low SES mirrored that for all beneficiaries, with linear RRs across the range of PM_2.5_ exposures for CVD and respiratory mortality and lower RRs for PM_2.5_ exposures for low PM_2.5_ exposures (< 10 *μg*/*m*^3^) (Figure S3d-e).

### Non-traffic PM_2.5_ and cause-specific mortality

We found our method to estimate non-traffic PM_2.5_ performed well, as evidenced by the lack of correlation between the residuals of PM_2.5_ on NO_2_ and EC, a marker of traffic-related PM_2.5_ (*r* = 0.02), in the subset of data at CSN monitoring sites. As a comparison, total PM_2.5_ was significantly correlated with EC (*r* = 0.27). Correlations of the residuals of PM_2.5_ on NO_2_ with sulfate were statistically significant (*r* = 0.70), but lower than that between total PM_2.5_ and sulfate (r=0.87), suggesting that the non-traffic PM_2.5_ may also reflect, albeit to a lesser degree, secondary PM_2.5_.

When RRs were estimated for non-traffic PM_2.5_, we found substantially lower RRs as compared to all PM_2.5_ for all causes of death in both base- and SES-adjusted models. In base models, RRs for non-traffic PM_2.5_ were significant and positive for all causes of death, except for CHF, COPD, and lung cancer, for which associations remained positive but were no longer statistically significant (Table 2). Associations were attenuated after adjustment for SES and were no longer statistically significant for any respiratory-related cause of death. As with all PM_2.5_, RRs for non-traffic PM_2.5_ were highest for CVD-related causes of death, equaling 1.027 (95% CI: 1.011,1.043) for IHD and 1.057 (95% CI: 1.029,1.085) for CBV mortality, although the RR for all CVD (1.016; 95% CI: 1.005,1.028) was similar to that for non-accidental (1.014; 95% CI: 1.007,1.021) mortality.

## Discussion

Our study is the first to examine the impacts of exposure to all PM_2.5_ and non-traffic related PM_2.5_ on specific causes of mortality for all US Medicare beneficiaries (65+ years), totaling 53 million older adults living in over 41,000 ZIP codes throughout the US, totaling 3.8 billion person-months of follow-up. By virtue of its near complete sample of US older adults, our study is also the first to show exposure-response curves for specific causes of death for non-white, low SES, and rural populations for whom the PM_2.5_-associated risks for specific causes of death are poorly understood, especially in comparison to white and urban populations. We were able to do so by virtue of our novel computational methods, which allowed us to analyze linear and non-linear associations for the entire population and for specific subpopulations, including understudied rural and minority beneficiaries. We found a 10 *μg* /m^3^ increase in 12-month average PM_2.5_ to be associated with a 5% increase in all-cause mortality, as well as an 8.8, 5.6, and 2.5% increase in all CVD-, all respiratory-, and all cancer-related deaths, respectively, in age, sex, race, ZIP code, and SES-adjusted models. PM_2.5_-associated risks were null for lung cancer. Risks were essentially unchanged after controlling for ozone. Risks associated with non-traffic PM_2.5_ were lower as compared to that for all PM_2.5_, with wider confidence intervals, but remained significant for all cause and CVD mortality and were null for respiratory and lung cancer-related deaths.

RRs for CVD- and respiratory-related mortality were linear, with statistically significant and positive RRs and no evidence of a threshold level below which PM_2.5_ was not associated with mortality. RRs for non-accidental and all cancer mortality also showed no evidence of a threshold level. For all cancer mortality, exposure-response curves were supra-linear, with higher RRs when PM_2.5_ was below as compared to above 10 *μg*/*m*^3^. The opposite pattern was found for non-accidental mortality; however, for both causes of death, RRs remained statistically significant and positive across the range of PM_2.5_ exposures. Importantly, our findings for non-accidental mortality (which comprise 97% of all deaths) are consistent with previous studies. Using a similar dataset but different analytical methods, Di et al. (2017) also found “almost linear*”* associations between PM_2.5_ and all-cause mortality, with no evidence of a threshold, only small deviations from linearity, and lower associations when PM_2.5_ exposures were below approximately 8 *μg*/*m*^3^. Notably, when our analyses were limited to urban areas, with or without control for ozone, PM_2.5_ exposure-response curves for non-accidental mortality were linear, suggesting that non-linearity in exposure-response curves may reflect different mortality risks for rural beneficiaries, who experienced lower mean PM_2.5_ exposures and lower RRs. Our findings for urban beneficiaries may explain the observed linear or supra-linear associations from previous US, Canadian and western European cohort studies, which were based primarily in urban areas. Note that our findings with regard to the shape of the exposure-response are not comparable to those reported in Cohen et al. [22] in their Global Burden of Disease analysis, which was intended to estimate effects of a wide range of PM_2.5_ exposure levels, including those well above that observed in US, Canadian, and western European air pollution cohort studies. Thus, the range of exposures examined in Cohen et al. [22] were substantially higher than that in our study.

We found increased mortality risks from long-term PM_2.5_ exposures to be strongest for CVD-related diseases, with significant and positive risks of increased mortality from all CVD, IHD, and CBV, but not CHF. While attenuated, our findings for all CVD, IHD, and CBV remained positive and significant upon adjustment for SES. In addition, CVD, IHD, and CBV mortality risks were similar across the range of PM_2.5_ exposures, with no evidence of a lower threshold for response or of smaller RRs at low PM_2.5_ levels. PM_2.5_-mortality associations for CVD-related causes were positive and significant for beneficiaries of different sexes, races, ages, and living in ZIP codes with different SES and urbanicities. They further are within the range of previously reported findings, with comparable RRs to those reported for American Cancer Society (ACS) [23], Roman, and Netherlands (NCLS) cohorts [24], higher risks than in our earlier study of Medicare beneficiaries living near air pollution monitors [4], and lower risks than reported in several North American cohort studies [2, 25, 26], including those focused on low PM_2.5_ concentrations [27, 28]. Our lower RRs may be due in part to our inclusion of understudied rural and minority beneficiaries, for whom RRs were lower, or may suggest that our models overcontrolled for SES.

We saw similarly robust associations between long-term PM_2.5_ and increased respiratory mortality, for which previous evidence has been mixed. As in our earlier study of Medicare beneficiaries living near air pollution monitors [4], 12-month PM_2.5_ exposures were significantly associated with increased mortality from all respiratory causes, COPD and pneumonia. Although we found neighborhood SES to confound the association, RRs for respiratory mortality remained positive and significant in SES-adjusted models. Further, PM_2.5_-associated risks of increased respiratory mortality were significant for all age, sex, SES, and most racial groups, but were null for Hispanic and non-urban beneficiaries. Our null finding for respiratory mortality within rural populations raise concerns about potential confounding by smoking. Although Di et al. [5] ruled out smoking as a potential confounder in their sensitivity analysis, their analysis focused only on all-cause mortality and further did not specifically assess confounding by smoking within rural populations, who have higher smoking rates [29] and lower average PM_2.5_ levels. As with CVD mortality, we found no evidence of a lower threshold for response, with linear exposure-response curves for mortality from all respiratory and COPD causes. For pneumonia mortality, higher RRs were found when PM_2.5_ exposures were below as compared to above 10 *μg* /m^3^, suggesting that linear models underestimate PM_2.5_-pneumonia mortality risks at low exposure levels.

Significant and positive associations between PM_2.5_ and all cancers but not lung cancer in SES adjusted models were somewhat unexpected, adding to mixed findings for lung cancer from earlier studies. While our base results are similar to those from our earlier study of Medicare beneficiaries living near air monitors [4], our non-significant SES-adjusted findings for lung cancer are consistent with null associations reported in several US and European cohort studies [30–34]. They, however, are contrary to that from a meta-analysis that showed significant PM_2.5_-associated lung cancer mortality risks [35], reflecting significant positive associations from several large-scale North American, European, and Asian cohort studies [23, 36–38].

Notably, we found mortality risks to be lower for non-traffic PM_2.5_ as compared to total PM_2.5_, with positive and significant associations for all cause and CVD-related mortality and null associations for respiratory and lung cancer-related deaths. While not examined explicitly, these findings suggest that traffic-related PM is a key contributor to the risks posed by PM_2.5_ for all cause and CVD mortality and the primary contributor to respiratory disease and cancer mortality. Alternatively or in addition, our findings may also indicate the importance of primary (but not secondary) PM_2.5_ as a risk factor for mortality, given the high correlation of non-traffic PM_2.5_ with sulfate. As a measure of non-traffic and traffic PM_2.5_-associated risks, however, our findings are consistent with previous long-term studies, including those from Netherlands Cohort Study on Diet and Cancer (NLCS) [30, 39], the Washington University–EPRI Veterans’ Cohort Mortality Study [40, 41], and the National Particle Component Toxicity Initiative (NPACT) study [42]. The NPACT study was novel in its examination of the mortality impacts of long-term PM_2.5_ from traffic as well as from other sources, finding strong associations between traffic and especially coal combustion source categories and increased IHD mortality [42]. These results differ somewhat from our findings. While we found significant associations of both total and non-traffic PM_2.5_ on CVD and IHD mortality, associations were comparatively stronger for total PM_2.5_ as compared to non-traffic PM_2.5_, which given its high correlation with sulfate, may also serve as a marker for secondary PM_2.5_ and/or coal-combustion. Differences in our findings likely result from imprecision in estimates of non-traffic PM_2.5_, as well as differences in our study population and control for confounders.

Across the examined causes of death, we provide compelling evidence that PM_2.5_-associated RRs differ by age, race, urbanicity, and to a lesser extent by SES. We found higher risks for older adults younger as compared to older than 75 years, likely reflecting the increasing baseline risk of death with age and the many competing risks for mortality at older ages [43]. For race, we found PM_2.5_-associated risks to be highest in Blacks for all causes of death, consistent with previous studies [11], followed by Whites for non-accidental and CVD mortality and by Asians for respiratory and cancer-related mortality. Notably, PM_2.5_-associated risks were null or protective for Asians for non-accidental and CVD-related mortality and for Hispanics for all causes of death. Protective associations for Hispanics were puzzling, possibly reflecting unmeasured confounding by social or economic factors and/or the very heterogeneous US Hispanic population, with regard to social, economic, geographic, and country of origin [44, 45]. This heterogeneity has been cited by MESA and other studies [46–48] as a possible factor explaining often null or even protective health findings for Hispanics. Clearly, additional studies are needed to examine these factors and their impact of long-term air pollution exposures on health risks in Hispanic and other minority groups.

Higher mortality risks in urban ZIP codes are consistent with findings showing combustion particles to comprise a larger fraction of PM_2.5_ in urban areas [49] and to be more toxic than non-combustion components [50]. For urban beneficiaries, the risks of non-accidental and CVD-related mortality were somewhat higher for individuals living in high as compared to low SES neighborhoods, perhaps indicative of a higher portion of combustion-related pollution in low SES neighborhoods [51].

Whether the higher PM_2.5_ exposures experienced by beneficiaries living in urban ZIP codes contribute to their higher RRs is not clear. For CVD- and respiratory-related mortality, the observed linear mortality risks across the range of PM_2.5_ concentrations suggests that it does not, as RRs for these causes of death did not depend on the magnitude of PM_2.5_ exposures. In contrast, we found lower RRs for non-accidental mortality for PM_2.5_ exposures less than 10 *μg* /m^3^, consistent with our finding of lower RRs for non-urban ZIP codes. As above, our null (and even significant but protective) RRs for lung cancer and respiratory mortality within rural populations may also reflect confounding by smoking [29]; however, potential confounding of associations between PM_2.5_ and lung cancer and respiratory mortality by smoking with rural populations has not yet been studied.

Our study had several limitations. First, we only had information on beneficiaries’ ZIP code of residence, contributing to exposure misclassification. We, however, accounted for beneficiaries’ residential moves and used validated spatio-temporal models to predict exposures, thus reducing error [52]. Previous studies found exposure error from imprecise spatial estimates to bias results towards the null, lending support to our findings of significant positive associations [14]. Second, we examined associations between PM_2.5_ and multiple mortality causes, raising multiple comparison concerns. These concerns are balanced by the consistency of our findings across outcomes and model specifications. Third, while we did not have data on personal-level characteristics, we allowed baseline hazards to vary by age, sex, race, and ZIP code and adjusted our models for ZIP code-level SES. Confounding by other personal-level characteristics is unlikely to explain our findings, given previous findings for urban populations showing little change in mortality risks after adjustment for individual-level characteristics [53] and given the high proportion of our population living in urban areas. However, it is possible that residual confounding, such as that from smoking, may introduce bias in the PM_2.5_-mortality associations among non-urban populations, offering a possible explanation for the lower RRs observed for these populations. Finally, NO_2_ serves as an imperfect proxy of traffic-related PM_2.5_. As a result, the mortality risks estimated using the residuals of NO_2_ on PM_2.5_ are an imperfect measure of non-traffic exposures, potentially contributing to our observed wider confidence intervals for the RRs for non-traffic PM_2.5_. Nevertheless, our results provide a first direct assessment of the mortality risks resulting from long-term exposures to non-traffic PM_2.5_. Importantly, our findings of lower mortality risks for non-traffic as compared to total PM_2.5_ are consistent with results from studies of long-term traffic exposures and mortality. However, additional studies are needed to assess consistency of findings using this approach.

These limitations are balanced by the substantial strengths of our study. With nearly 53 million Medicare beneficiaries, 16 million deaths, and complete coverage of Medicare beneficiaries living across the US, our analyses were well-powered to detect meaningful associations, allowing us to provide valuable new information on the impact of long-term PM_2.5_ exposures on specific causes of CVD, respiratory, and cancer-related deaths at low PM_2.5_ levels and for previously under-studied minority and rural populations. We were thus able to demonstrate linearity in exposure-response curves for CVD and respiratory mortality and disproportionate PM_2.5_-associated deaths in beneficiaries who are Black and to a lesser extent Asian, are of low SES, or live in urban neighborhoods.

## Conclusions

With nearly 53 million older Medicare beneficiaries living across the U.S., we observed that PM_2.5_ was associated with mortality from cardiovascular, respiratory and all cancer disease, but not lung cancer. PM_2.5_-associated risks of CVD and respiratory mortality were similar across PM_2.5_ levels, with no evidence of a threshold. Blacks, urban, and younger beneficiaries were most vulnerable to the long-term impacts of PM_2.5_ on mortality.

## Supplementary information

**Additional file 1: Appendix S1.** Cox PH for Large-scale data. **Table S1.** Validation of Java implementation of Cox PH models using public package in R. **Table S2.** Percent of deaths by cause and beneficiary characteristics. **Table S3.** Association of long-term PM_2.5_ and cause-specific mortality, with and without control for ozone (O_3_). **Table S4.** Association of long-term PM_2.5_ and cause-specific mortality for low PM_2.5_ ZIP Code. **Table S5**. Linear Effect Modification Analysis by cause of death. **Figure S1.** SES-adjusted exposure-response curves for 12-month average PM_2.5_ and specific causes of death. **Figure S2.** Sensitivity analyses: (a) SES-adjusted and (b) SES- and ozone-adjusted exposure-response curves for 12-month moving average PM_2.5_ and all-cause, CVD, respiratory and cancer deaths. **Figure S3.** SES-adjusted exposure-response curves for 12-month average PM_2.5_ and cause-specific mortality by effect modifier. **Figure S4.** SES-adjusted exposure-response curves for 12-month average PM_2.5_ and all causes mortality with different number of knots. **Figure S5.** SES-adjusted exposure-response curves for 12-month average PM_2.5_ and all causes mortality near monitoring sites.

## Data Availability

Java implementation of Cox Proportional Hazards model is available on GitHub (https://github.com/Rainicy/survival) and our PM_2.5_ exposure estimates for each Zip Code available upon request.

## Abbreviations

ACS: American Cancer Society
CBV: Cerebrovascular Disease
CHF: Congestive Heart Failure
CI: Confidence Interval
COPD: Chronic Obstructive Pulmonary Disease
Cox PH: Cox Proportional Hazards
CSN: Chemical speciation network
CVD: Cardiovascular Disease
EC: Elemental Carbon
EPA: Environmental Protection Agency
ICD: International Classification of Disease
IHD: Ischemic Heart Disease
IRS: Internal Revenue Service
NO_2_: Nitrogen Dioxide
PM_2.5_: Particulate matter with aerodynamic diameter of 2.5 *μm* or less
ppb: Parts per Billion
RCS: Restricted Cubic Splines
RHRC: Rural Health Research Center
RRs: Risk Ratios
SES: Socio-Economic Status
U.S.: United States
