# Supplementary material for: The impact of long-term PM2.5 exposure on specific causes of death: exposure-response curves and effect modification among 53 million U.S. Medicare beneficiaries
Source: Environ Health. 2020 Feb 17;19:20. doi: 10.1186/s12940-020-00575-0 (PMC7026980; doi:10.1186/s12940-020-00575-0)
Supplement: Supplementary file 1 — Additional file 1: Appendix S1. Cox PH for Large-scale data. Table S1. Validation of Java implementation of Cox PH models using public package in R. Table S2. Percent of deaths by cause and beneficiary characteristics. Table S3. Association of long-term PM2.5 and cause-specific mortality, with and without control for ozone (O3). Table S4. Association of long-term PM2.5 and cause-specific mortality for low PM2.5 ZIP Code. Table S5. Linear Effect Modification Analysis by cause of death. Figure S1. SES-adjusted exposure-response curves for 12-month average PM2.5 and specific causes of death. Figure S2. Sensitivity analyses: (a) SES-adjusted and (b) SES- and ozone-adjusted exposure-response curves for 12-month moving average PM2.5 and all-cause, CVD, respiratory and cancer deaths. Figure S3. SES-adjusted exposure-response curves for 12-month average PM2.5 and cause-specific mortality by effect modifier. Figure S4. SES-adjusted exposure-response curves for 12-month average PM2.5 and all causes mortality with different number of knots. Figure S5. SES-adjusted exposure-response curves for 12-month average PM2.5 and all causes mortality near monitoring sites. [file 12940_2020_575_MOESM1_ESM.docx]

**Supplemental Material**

**The Impact of Long-Term PM_2.5_ Exposure on Specific Causes of Death: Exposure-Response Curves and Effect Modification among 53 million U.S. Medicare Beneficiaries**

Bingyu Wang; Ki-Do Eum; Fatemeh Kazemiparkouhi; Cheng Li; Justin Manjourides; Virgil Pavlu; Helen Suh

Table of Contents

[Appendix S1. Cox PH for Large-Scale Data 3](#_Toc23163231)

[Table S1. Validation of Java implementation of Cox PH models using public package in R 7](#_Toc23163232)

[Table S2. Percent of deaths by cause and beneficiary characteristics 8](#_Toc23163233)

[Table S3. Association of long-term PM_2.5_ and cause-specific mortality, with and without control for ozone (O_3_) 9](#_Toc23163234)

[Table S4. Association of long-term PM_2.5_ and cause-specific mortality for low PM_2.5_ ZIP Code 10](#_Toc23163235)

[Table S5. Linear Effect Modification Analysis by cause of death 11](#_Toc23163236)

[Figure S1. SES-adjusted exposure-response curves for 12-month moving average PM_2.5_ and specific causes of death 16](#_Toc23163237)

[(a) Cardiovascular-related diseases 16](#_Toc23163238)

[(b) Respiratory-related diseases 17](#_Toc23163239)

[(c) Cancer and Lung Cancer 18](#_Toc23163240)

[Figure S2. Sensitivity analyses: (a) SES-adjusted and (b) SES- and ozone-adjusted exposure-response curves for 12-month moving average PM_2.5_ and all-cause, CVD, respiratory and cancer deaths 19](#_Toc23163241)

[(a) SES-Adjusted models (no control for ozone) 19](#_Toc23163242)

[(b) SES- and ozone-adjusted models 20](#_Toc23163243)

[Figure S3. SES-adjusted exposure-response curves for 12-month moving average PM_2.5_ and cause-specific mortality by effect modifier 21](#_Toc23163244)

[(a) Sex 21](#_Toc23163245)

[(b) Race 22](#_Toc23163246)

[(c) Age 23](#_Toc23163247)

[(d) Urbanicity 24](#_Toc23163248)

[(e) SES 25](#_Toc23163249)

[Figure S4. SES-adjusted exposure-response curves for 12-month moving average PM_2.5_ and all causes mortality with different number of knots 26](#_Toc23163250)

[Figure S5. SES-adjusted exposure-response curves for 12-month moving average PM_2.5_ and all causes mortality near monitoring sites. 27](#_Toc23163251)

[References 28](#_Toc23163252)

# Appendix S1. Cox PH for Large-Scale Data

To perform our analyses of 12-month PM_2.5_ exposures and cause-specific mortality on our large-scale data, we implemented both linear and non-linear Cox PH methods in Java. Our implementation overcame memory and processing limitations of conventional software packages, such as R and SAS, using data grouping and linkage methods, optimization techniques, and multi-threading, as detailed below. Our Java implementation will be hosted on GitHub once our paper is accepted. These methods were able to run our models that included data for 53 million Medicare beneficiaries and 3.8 billion person-months of follow-up in approximately ten minutes. Below we briefly present our data grouping methods, followed by our Cox PH re-implementation for the linear model, then the restricted cubic spline, and finally their validation.

- **Data Processing.** We created joint datasets that minimize redundant entries by aggregating rows with the same attributes by ZIP code and month and by adding to these joint datasets two counting attributes: the number of deaths and the total population for the ZIP code and month.
- **Re-Implementation of Linear Cox PH model.** The original Cox PH estimates air pollution-associated mortality risks using stratum-specific baselines (1):

$$h\left( t_{i} | X_{i}, s_{i} \right)={h_{0}}_{s}\exp\left( \boldsymbol{\beta}^{T}\boldsymbol{x}_{i} \right)$$

where $i\in[1, n]$ represents each individual $i$, with $n$ the total number of individuals. ${h_{0}}_{s}$ is a stratum-specific baseline hazard function, $y_{i} = min(t_{i}, c_{i}),$where $t_{i}$ is event time and $c_{i}$the right-censoring time for each individual $i$. In addition, let $\boldsymbol{x}_{i}=\left( x_{i1}, x_{i2}, \ldots, x_{ip} \right)^{T}$ be a p-vector of covariates for the individual $i$, and $\boldsymbol{\beta}=\left( \beta_{1}, \beta_{2}, \ldots, \beta_{p} \right)^{T}$ be the p-vector of estimating model parameters. The $n$ observed data $D=\{\left( y_{i}, \delta_{i}, \boldsymbol{x}_{\boldsymbol{i}} \right):i\in[1,n]\}$, where $\delta_{i}=I(t_{i}\leq c_{i})$ is an indicator variable such that $\delta_{i}=1$ if the observation is not censored and 0 otherwise.

To simplify the problem, a partial likelihood function of Cox PH was proposed by (1):

$$L_{p}\left( \boldsymbol{\beta} | D \right)=\prod_{i=1}^{n} \left( \frac{\exp\left( \boldsymbol{\beta}^{\boldsymbol{T}}\boldsymbol{x}_{\boldsymbol{i}} \right)}{\sum_{t\in R\left( y_{i} \right)} \exp\left( \boldsymbol{\beta}^{\boldsymbol{T}}\boldsymbol{x}_{\boldsymbol{t}} \right)} \right)^{\delta_{i}}$$

where $R(y_{i})$ is the risk set of the given individual $i$, and $R\left( y_{i} \right)=\{t:y_{t}\geq y_{i}\}$, representing any individual $t$, who has survived at least longer than individual $i$. In general, we assumed no tied survival times, adding a very small random number (uniform from [$-{10}^{-5}, {10}^{-5}$]) to the event time (2). Since the survival time order, but not actual event time, is used when updating the model, we simply shuffle the risk set and end up with ordered event times to break ties in our implementation. Furthermore, optimizing the above partial likelihood is equivalent to estimate the partial log-likelihood function:

$$l_{p}\left( \beta\right|D)= \sum_{i=1}^{n} \delta_{i}\left\{ \boldsymbol{\beta}^{\boldsymbol{T}}\boldsymbol{x}_{\boldsymbol{i}}-\log\left[ \sum_{t\in R\left( y_{i} \right)} \exp\left( \boldsymbol{\beta}^{\boldsymbol{T}}\boldsymbol{x}_{\boldsymbol{t}} \right) \right] \right\} [1]$$

To simplify model interpretation, we omit the penalty term of $\boldsymbol{\beta}$; however, the L2 penalty term has been adopted in our Cox PH model. It is worth noting that Eq [1], even with the L2 penalty term, is a convex function, and a wide range of optimization algorithms can be utilized. We chose to employ the Limited-memory BFGS (L-BFGS) algorithm (3) to minimize the negative log-partial likelihoods:

$$\boldsymbol{\beta}^{*}=argmin_{\boldsymbol{\beta}}-l_{p}(\boldsymbol{\beta}|D)$$

L-BFGS is a limited memory quasi-Newton methods for large scale optimization, which has been employed as one of the most popular optimization algorithms in machine learning (4). As a quasi-Newton method, L-BFGS is not required to compute the Hessian matrix of variables, but to estimate an approximation of Hessian with only a few vectors. Thus, the L-BFGS method is particularly well suited for optimization problems with a large number of variables. To simplify computation, we grouped subjects that share the same variables ($\boldsymbol{x}_{g}$), with two counts for each group $g$: number of deaths ($\delta_{g}$) and total number of people ($y_{g}$), and redefined Eq [1] as:

$$l_{p}\left( \beta\right|D, G)= \sum_{g\in G} \delta_{g}\left\{ \boldsymbol{\beta}^{\boldsymbol{T}}\boldsymbol{x}_{\boldsymbol{g}}-\log\left[ \sum_{t\in R\left( y_{g} \right)} y_{g}\exp\left( \boldsymbol{\beta}^{\boldsymbol{T}}\boldsymbol{x}_{\boldsymbol{t}} \right) \right] \right\} [2]$$

where $G$ is the total distinct groups from the whole dataset, which given strata for sex (male, female), race (black, white, hispanic, asian, other), age (26 age categories) and ZIP code (41,630), with each ZIP code having distinct PM_2.5_ concentrations, we have over 10 milliondistinct groups at any given time point. The partial log-likelihood Eq [2] can be considered as one component from a specific strata or group $G$:

$$l_{p}\left( \beta| D, S \right)= \sum_{G\in S} l_{p}\left( \beta| D, G \right)$$

which is still a convex optimization problem.

- **Restricted Cubic Splines.** To study the non-linear relationship between the cause of death and exposures, we implemented restricted cubic splines (RCS) with 3 knots (5) in Cox PH models. RCS divides the continuous range of predictor variables with a few number ($l$) of pre-defined "knots", which are written as $k_{1}, k_{2}, \ldots, k_{l}$ in an ascending order. With $l$ knots, a size of $l-2$ new variables will be generated for each original variable ($x$) by the following formula:

$x^{i}=\left( x-k_{i} \right)_{+}^{3}-\left( x-k_{l-1} \right)_{+}^{3}\frac{k_{l}-k_{i}}{k_{l}-k_{l-1}}+\left( x-k_{l} \right)_{+}^{3}\frac{k_{l-1}-k_{i}}{k_{l}-k_{l-1}}$[3]

For $i=1, 2, \ldots, l-2$. And $u_{+}$is defined as

$$u_{+}= \left\{ \begin{aligned} u if u> 0 \\ 0 otherwise \end{aligned} \right.$$

We further applied the $norm=2$ settings (6), which normalizes each new variable by the two-thirds of the spacing between the first and last knots:

$$x_{norm}^{i}=\frac{x^{i}}{\left( k_{l}-k_{1} \right)^{\boldsymbol{2/3}}}$$

wherein $x^{i}$ is calculated from Eq [3]. The normalization transforms all new generated non-linear terms to the original variable scale (6).

- **Model Validation.** To verify of our Java implementation of the stratified Cox PH model with and without restricted cubic splines, we created a sample dataset of 10,000 subjects, which is sufficiently small to run our full analysis in R. For each subject, we included data on age (65-90), gender (male or female), race (White, Black, Hispanic, Asian, other), location (800 sites), date (120 total months), and death (0 or 1), totaling 1,011,945 subject-date records. We applied the Cox PH function **coxph** from the "**survival**" package in R (7), with strata on age, gender and race, and Restricted Cubic Splines for the exposure using **rcspline.eval** from "**Hmisc**" package in R (6). Knots were specified at the 10^th^, 50^th^, and 90^th^ percentiles. We also used the same settings in our Java implementation of these models and compared our findings to those from R. As shown in Table S1, our Java implementation provided almost identical risk estimates and standard errors to those obtained in R.

# Table S1. Validation of Java implementation of Cox PH models using public package in R

| Estimators | Linear Cox PH | | Non-Linear Cox PH | |
| --- | --- | --- | --- | --- |
|  | R | Java | R | Java |
| $\beta_{x}$  (se) | 0.00906 (0.00895) | 0.00904 (0.00895) | 0.00948  (0.02367) | 0.00940  (0.02367) |
| $\beta_{x_{norm}^{1}}$  (se) | - | - | 0.00197  (0.01982) | 0.00205  (0.01982) |
| Both linear and non-linear models include strata for age, gender, race, and ZIP code. Non-linear models are estimated using restricted cubic splines with 3 knots. | | | | |

# Table S2. Percent of deaths by cause and beneficiary characteristics

| Factor | All | Non-Accid | Accid-ental | CVD | IHD | CBV | CHF | Resp | COPD | Pneu | Cancer | Lung Cancer |
| --- | --- | --- | --- | --- | --- | --- | --- | --- | --- | --- | --- | --- |
| Gender  Male | 44.8 | 44.4 | 53.5 | 43.0 | 47.1 | 36.7 | 37.6 | 46.7 | 48.0 | 42.9 | 51.4 | 56.0 |
| Female | 55.2 | 55.6 | 46.5 | 57.0 | 52.9 | 63.3 | 62.4 | 53.3 | 52.0 | 57.1 | 48.6 | 44.0 |
| Race  White | 87.0 | 87.3 | 89.7 | 87.0 | 87.4 | 86.3 | 89.5 | 90.5 | 92.5 | 87.6 | 87.1 | 88.5 |
| Non-white | 13.0 | 12.7 | 10.3 | 13.0 | 12.6 | 13.7 | 10.5 | 9.5 | 7.5 | 12.4 | 12.9 | 11.5 |
| Age  >75 | 75.9 | 76.0 | 71.9 | 80.7 | 79.1 | 83.6 | 88.0 | 76.9 | 71.0 | 87.2 | 62.3 | 53.7 |
| $\leq$75 | 24.1 | 24.0 | 28.1 | 19.3 | 20.9 | 16.4 | 12.0 | 23.1 | 29.0 | 12.8 | 37.7 | 46.3 |
| Urbanicity  Urban | 74.0 | 74.0 | 70.5 | 73.9 | 75.0 | 72.8 | 69.5 | 72.8 | 72.3 | 73.3 | 74.6 | 74.0 |
| Non-urban | 26.0 | 26.0 | 29.5 | 26.1 | 25.0 | 27.2 | 30.5 | 27.2 | 27.7 | 26.7 | 25.4 | 26.0 |
| SES  Low | 12.1 | 12.0 | 11.8 | 11.8 | 12.0 | 10.7 | 11.2 | 11.6 | 11.9 | 11.4 | 12.2 | 12.4 |
| Middle | 52.9 | 52.9 | 53.2 | 53.3 | 53.7 | 52.6 | 54.0 | 53.8 | 55.0 | 52.4 | 52.7 | 54.3 |
| High | 35.1 | 35.1 | 35.0 | 34.9 | 34.2 | 36.7 | 34.7 | 34.6 | 33.1 | 36.2 | 35.1 | 33.3 |
| Abbreviations: CVD (all Cardiovascular), IHD (Ischemic heart disease), CBV (Cerebrovascular disease), CHF (Congestive heart failure), Resp (all Respiratory), COPD (Chronic Obstructive Pulmonary Disease), Pneu (Pneumonia).  Medicare enrollees aged 65-120 Years, United States, from 2000 to 2008. | | | | | | | | | | | | |

# Table S3. Association of long-term PM_2.5_ and cause-specific mortality, with and without control for ozone (O_3_)

| Cause of Death | O_3_ Adjusted | | | | | | | | | Non-O_3_ Adjusted | | | | | |
| --- | --- | --- | --- | --- | --- | --- | --- | --- | --- | --- | --- | --- | --- | --- | --- |
|  | Base Model | | | | | SES-Adjusted | | | | Base Model | | | SES-Adjusted Model | | |
|  | **RR** | **95% CI** | | | **RR** | | | **95% CI** | | **RR** | **95% CI** | | **RR** | **95% CI** | |
| All Cause | 1.236 | 1.227 | 1.245 | 1.051 | | | 1.042 | | 1.060 | 1.241 | 1.232 | 1.250 | 1.052 | 1.043 | 1.061 |
| Non-accidental | 1.246 | 1.236 | 1.255 | 1.052 | | | 1.043 | | 1.062 | 1.250 | 1.241 | 1.260 | 1.053 | 1.044 | 1.063 |
| All Cardiovascular | 1.638 | 1.620 | 1.657 | 1.090 | | | 1.076 | | 1.105 | 1.650 | 1.632 | 1.669 | 1.091 | 1.076 | 1.106 |
| IHD | 1.904 | 1.875 | 1.934 | 1.140 | | | 1.119 | | 1.161 | 1.919 | 1.890 | 1.949 | 1.140 | 1.119 | 1.161 |
| CBV | 1.909 | 1.857 | 1.962 | 1.129 | | | 1.094 | | 1.166 | 1.925 | 1.874 | 1.978 | 1.129 | 1.094 | 1.166 |
| CHF | 0.980 | 0.935 | 1.026 | 0.866 | | | 0.821 | | 0.915 | 0.987 | 0.942 | 1.034 | 0.866 | 0.821 | 0.915 |
| All Respiratory | 1.258 | 1.231 | 1.286 | 1.050 | | | 1.023 | | 1.078 | 1.261 | 1.233 | 1.289 | 1.050 | 1.023 | 1.078 |
| COPD | 1.070 | 1.037 | 1.104 | 1.014 | | | 0.978 | | 1.051 | 1.069 | 1.037 | 1.102 | 1.016 | 0.980 | 1.053 |
| Pneumonia | 1.696 | 1.631 | 1.764 | 1.083 | | | 1.032 | | 1.135 | 1.709 | 1.644 | 1.777 | 1.082 | 1.032 | 1.135 |
| All Cancer | 1.148 | 1.130 | 1.167 | 1.013 | | | 0.994 | | 1.032 | 1.150 | 1.132 | 1.169 | 1.013 | 0.994 | 1.032 |
| Lung cancer | 1.079 | 1.046 | 1.113 | 0.986 | | | 0.951 | | 1.023 | 1.082 | 1.049 | 1.116 | 0.987 | 0.952 | 1.023 |

Abbreviation: RR, Risk Ratio, associated with 10$\mu g/m^{3}$ increase in PM_2.5_ concentration; 95% CI, 95% lower and upper confidence interval; IHD, ischemic heart disease; CBV, cerebrovascular disease; CHF, congestive heart failure; COPD, chronic obstructive pulmonary disease.

Cox Proportional Hazard base models fit with strata for gender, race, age, and ZIP code. SES adjusted model additionally adjusts for ZIP code-level SES. Analyses based on subset of data, including over 22 million beneficiaries living within 6 miles of an EPA ozone monitor.

# Table S4. Association of long-term PM_2.5_ and cause-specific mortality for low PM_2.5_ ZIP Code

| Cause of Death | <8$\boldsymbol{\mu g/}\boldsymbol{m}^{\boldsymbol{3}}$ | | | <10$\boldsymbol{\mu g/}\boldsymbol{m}^{\boldsymbol{3}}$ | | | <12$\boldsymbol{\mu g/}\boldsymbol{m}^{\boldsymbol{3}}$ | | |
| --- | --- | --- | --- | --- | --- | --- | --- | --- | --- |
|  | RR | 95% CI | | RR | 95% CI | | RR | 95% CI | |
| All Causes | 1.106 | 1.089 | 1.123 | 1.167 | 1.156 | 1.178 | 1.208 | 1.200 | 1.217 |
| Non-accidental | 1.114 | 1.097 | 1.132 | 1.175 | 1.164 | 1.187 | 1.217 | 1.208 | 1.225 |
| Accidental | 0.863 | 0.786 | 0.948 | 0.865 | 0.816 | 0.918 | 0.885 | 0.846 | 0.925 |
| All Cardiovascular | 1.336 | 1.303 | 1.369 | 1.533 | 1.510 | 1.556 | 1.659 | 1.641 | 1.677 |
| Ischemic heart disease | 1.500 | 1.449 | 1.553 | 1.685 | 1.723 | 1.796 | 1.967 | 1.937 | 1.998 |
| Cerebrovascular disease | 1.484 | 1.404 | 1.569 | 1.751 | 1.692 | 1.812 | 1.938 | 1.889 | 1.988 |
| Congestive heart failure | 0.975 | 0.888 | 1.070 | 1.155 | 1.093 | 1.221 | 1.170 | 1.124 | 1.218 |
| All Respiratory | 1.172 | 1.121 | 1.225 | 1.202 | 1.169 | 1.235 | 1.226 | 1.201 | 1.251 |
| COPD | 1.079 | 1.017 | 1.145 | 1.031 | 0.993 | 1.070 | 1.016 | 0.988 | 1.045 |
| Pneumonia | 1.559 | 1.422 | 1.709 | 1.685 | 1.595 | 1.780 | 1.887 | 1.812 | 1.966 |
| All Cancer | 1.093 | 1.058 | 1.129 | 1.134 | 1.112 | 1.157 | 1.154 | 1.137 | 1.171 |
| Lung cancer | 1.019 | 0.958 | 1.084 | 1.033 | 0.995 | 1.072 | 1.049 | 1.020 | 1.079 |
| Abbreviation: RR, Risk Ratio, associated with 10$\boldsymbol{\mu g/}\mathbf{m}^{\mathbf{3}}$ increase in PM_2.5_ concentration; 95% CI, 95% lower and upper confidence interval; COPD, Chronic Obstructive Pulmonary Disease.  Cox Proportional Hazard models fit with strata for gender, race, age, and ZIP code for ZIP codes with average PM_2.5_ concentrations over the study period (2000-2008) below 8, 10 or 12 $\boldsymbol{\mu g/}\mathbf{m}^{\mathbf{3}}$. | | | | | | | | | |

# Table S5. Linear Effect Modification Analysis by cause of death

| Causes of Death | No. of Death | Mean  ($\boldsymbol{\mu g/}\boldsymbol{m}^{\boldsymbol{3}}$) | RR (95% CI) |
| --- | --- | --- | --- |
| Non-Accidental | 15,324,059 | 10.55 | 1.051 (1.045,1.057) |
| Male | 6,797,991 | 10.45 | 1.074 (1.067,1.080) |
| Female | 8,526,068 | 10.62 | 1.039 (1.033,1.046) |
| White | 13,378,682 | 10.35 | 1.064 (1.058,1.071) |
| Non-white | 1,945,377 | 11.85 | 1.041 (1.034,1.049) |
| >75 | 11,640,482 | 10.59 | 1.003 (0.997,1.009) |
| $\leq$75 | 3,683,577 | 10.52 | 1.165 (1.158,1.173) |
| Urban | 11,343,972 | 11.12 | 1.066 (1.059,1.073) |
| Non-urban | 3,980,087 | 8.86 | 0.989 (0.977,1.002) |
| High income | 5,318,433 | 10.71 | 1.072(1.064,1.079) |
| Medium income | 8,009,505 | 10.36 | 1.048(1.041,1.054) |
| Low income | 1,816,628 | 11.03 | 1.031(1.022,1.041) |
| All Cardiovascular | 6,371,713 | 10.55 | 1.088 (1.078,1.098) |
| Male | 2,742,752 | 10.45 | 1.095 (1.084,1.105) |
| Female | 3,628,961 | 10.62 | 1.090 (1.080,1.100) |
| White | 5,543,750 | 10.35 | 1.109 (1.099,1.119) |
| Non-white | 827,963 | 11.85 | 1.054 (1.042,1.066) |
| >75 | 5,144,374 | 10.59 | 1.032 (1.022,1.041) |
| $\leq$75 | 1,227,339 | 10.52 | 1.252 (1.239,1.265) |
| Urban | 4,709,408 | 11.12 | 1.090 (1.079,1.101) |
| Non-urban | 1,662,305 | 8.86 | 1.078 (1.058,1.099) |
| High income | 2,199,606 | 10.71 | 1.116(1.104,1.128) |
| Medium income | 3,361,527 | 10.36 | 1.081(1.071,1.091) |
| Low income | 742,792 | 11.03 | 1.082(1.066,1.098) |

| Causes of Death | No. of Death | Mean  ($\boldsymbol{\mu g/}\boldsymbol{m}^{\boldsymbol{3}}$) | RR (95% CI) |  |
| --- | --- | --- | --- | --- |
| Ischemic heart disease | 3,323,527 | 10.55 | 1.126 (1.112,1.140) | |
| Male | 1,567,009 | 10.45 | 1.089 (1.075,1.103) | |
| Female | 1,756,518 | 10.62 | 1.167 (1.152,1.182) | |
| White | 2,906,288 | 10.35 | 1.154 (1.139,1.168) | |
| Non-white | 417,239 | 11.85 | 1.062 (1.045,1.079) | |
| >75 | 2,627,625 | 10.59 | 1.081 (1.068,1.095) | |
| $\leq$75 | 695,902 | 10.52 | 1.213 (1.196,1.230) | |
| Urban | 2,492,781 | 11.12 | 1.119 (1.104,1.134) | |
| Non-urban | 830,746 | 8.86 | 1.156 (1.125,1.188) | |
| High income | 1,126,251 | 10.71 | 1.152(1.136,1.170) | |
| Medium income | 1,767,676 | 10.36 | 1.118(1.103,1.133) | |
| Low income | 395,685 | 11.03 | 1.129(1.107,1.152) | |
| Cerebrovascular disease | 1,147,050 | 10.55 | 1.126 (1.103,1.150) | |
| ­­ Male | 421,456 | 10.45 | 1.177 (1.151,1.204) | |
| Female | 725,594 | 10.62 | 1.108 (1.084,1.132) | |
| White | 990,107 | 10.35 | 1.144 (1.119,1.168) | |
| Non-white | 156,943 | 11.85 | 1.102 (1.073,1.132) | |
| >75 | 959,318 | 10.59 | 1.067 (1.044,1.090) | |
| $\leq$75 | 187,732 | 10.52 | 1.374 (1.340,1.408) | |
| Urban | 834,741 | 11.12 | 1.140 (1.114,1.167) | |
| Non-urban | 312,309 | 8.86 | 1.073 (1.026,1.122) | |
| High income | 417,060 | 10.71 | 1.161(1.132,1.190) | |
| Medium income | 597,373 | 10.36 | 1.116(1.091,1.142) | |
| Low income | 121,043 | 11.03 | 1.134(1.095,1.174) | |

| Causes of Death | No. of Death | Mean  ($\boldsymbol{\mu g/}\boldsymbol{m}^{\boldsymbol{3}}$) | RR (95% CI) |
| --- | --- | --- | --- |
| Congestive heart failure | 471,127 | 10.55 | 0.986 (0.953,1.021) |
| Male | 176,910 | 10.45 | 1.019 (0.983,1.057) |
| Female | 294,217 | 10.62 | 0.976 (0.942,1.010) |
| White | 421,639 | 10.35 | 0.997 (0.963,1.032) |
| Non-white | 49,488 | 11.85 | 0.978 (0.934,1.024) |
| >75 | 414,413 | 10.59 | 0.930 (0.899,0.963) |
| $\leq$75 | 56,714 | 10.52 | 1.315 (1.261,1.372) |
| Urban | 327,297 | 11.12 | 0.938 (0.903,0.975) |
| Non-urban | 143,830 | 8.86 | 1.153 (1.080,1.232) |
| High income | 161,629 | 10.71 | 0.996(0.956,1.037) |
| Medium income | 251,737 | 10.36 | 0.992(0.956,1.028) |
| Low income | 52,389 | 11.03 | 0.955(0.904,1.009) |
| All Respiratory | 1,777,076 | 10.55 | 1.056 (1.038,1.074) |
| Male | 829,463 | 10.45 | 1.066 (1.047,1.085) |
| Female | 947,613 | 10.62 | 1.054 (1.036,1.073) |
| White | 1,608,972 | 10.35 | 1.059 (1.041,1.077) |
| Non-white | 168,104 | 11.85 | 1.066 (1.041,1.090) |
| >75 | 1,367,163 | 10.59 | 1.034 (1.016,1.052) |
| $\leq$75 | 409,913 | 10.52 | 1.075 (1.054,1.095) |
| Urban | 1,293,378 | 11.12 | 1.086 (1.066,1.107) |
| Non-urban | 483,698 | 8.86 | 0.942 (0.908,0.976) |
| High income | 607,284 | 10.71 | 1.087(1.065,1.109) |
| Medium income | 944,987 | 10.36 | 1.045(1.027,1.064) |
| Low Income | 203,520 | 11.03 | 1.072(1.044,1.102) |

| Causes of Death | No. of Death | Mean  ($\boldsymbol{\mu g/}\boldsymbol{m}^{\boldsymbol{3}}$) | RR (95% CI) |
| --- | --- | --- | --- |
| COPD | 944,665 | 10.55 | 1.023 (0.999,1.047) |
| Male | 453,690 | 10.45 | 1.030 (1.005,1.055) |
| Female | 490,975 | 10.62 | 1.024 (1.000,1.049) |
| White | 874,160 | 10.35 | 1.028 (1.004,1.052) |
| Non-white | 70,505 | 11.85 | 1.026 (0.993,1.061) |
| >75 | 670,795 | 10.59 | 0.997 (0.973,1.020) |
| $\leq$75 | 273,870 | 10.52 | 1.070 (1.043,1.097) |
| Urban | 683,292 | 11.12 | 1.047 (1.021,1.075) |
| Non-urban | 261,373 | 8.86 | 0.931 (0.886,0.978) |
| High income | 308,402 | 10.71 | 1.052(1.024,1.082) |
| Medium income | 513,420 | 10.36 | 1.010(0.985,1.035) |
| Low income | 110,842 | 11.03 | 1.053(1.014,1.092) |
| Pneumonia | 462,736 | 10.55 | 1.078 (1.044,1.114) |
| Male | 198,597 | 10.45 | 1.110 (1.073,1.148) |
| Female | 264,139 | 10.62 | 1.058 (1.024,1.094) |
| White | 405,238 | 10.35 | 1.084 (1.049,1.119) |
| Non-white | 57,498 | 11.85 | 1.058 (1.015,1.102) |
| >75 | 403,352 | 10.59 | 1.027 (0.995,1.061) |
| $\leq$75 | 59,384 | 10.52 | 1.244 (1.196,1.294) |
| Urban | 338,995 | 11.12 | 1.099 (1.061,1.139) |
| Non-urban | 123,741 | 8.86 | 0.990 (0.924,1.062) |
| High income | 165,755 | 10.71 | 1.104(1.062,1.147) |
| Medium income | 240,009 | 10.36 | 1.068(1.032,1.105) |
| Low income | 52,089 | 11.03 | 1.115(1.058,1.175) |

| Causes of Death | No. of  Death | Mean  ($\boldsymbol{\mu g/}\boldsymbol{m}^{\boldsymbol{3}}$) | RR (95% CI) |
| --- | --- | --- | --- |
| Cancer | 3,576,207 | 10.55 | 1.025 (1.012,1.038) |
| Male | 1,836,468 | 10.45 | 1.048 (1.035,1.062) |
| Female | 1,739,739 | 10.62 | 1.006 (0.993,1.019) |
| White | 3,114,300 | 10.35 | 1.031 (1.018,1.044) |
| Non-white | 461,907 | 11.85 | 1.048 (1.032,1.065) |
| >75 | 2,229,669 | 10.59 | 0.986 (0.974,0.999) |
| $\leq$75 | 1,346,538 | 10.52 | 1.092 (1.078,1.106) |
| Urban | 2,669,312 | 11.12 | 1.031 (1.018,1.045) |
| Non-urban | 906,895 | 8.86 | 0.997 (0.971,1.024) |
| High income | 1,241,856 | 10.71 | 1.042(1.027,1.057) |
| Medium income | 1,863,586 | 10.36 | 1.024(1.011,1.038) |
| Low income | 430,193 | 11.03 | 0.999(0.980,1.019) |
| Lung cancer | 988,643 | 10.55 | 0.995 (0.972,1.018) |
| Male | 553,337 | 10.45 | 1.061 (1.035,1.086) |
| Female | 435,306 | 10.62 | 0.923 (0.901,0.946) |
| White | 875,280 | 10.35 | 0.991 (0.968,1.014) |
| Non-white | 113,363 | 11.85 | 1.082 (1.050,1.116) |
| >75 | 531,384 | 10.59 | 0.960 (0.937,0.984) |
| $\leq$75 | 457,259 | 10.52 | 1.051 (1.025,1.076) |
| Urban | 731,175 | 11.12 | 1.010 (0.984,1.036) |
| Non-urban | 257,468 | 8.86 | 0.936 (0.891,0.983) |
| High income | 325,423 | 10.71 | 1.021(0.993,1.050) |
| Medium income | 530,507 | 10.36 | 0.991(0.967,1.015) |
| Low income | 121,504 | 11.03 | 0.973(0.938,1.009) |
| Abbreviation: RR, Risk Ratio, associated with 10$\mu g/m^{3}$ increase in PM_2.5_ concentration; 95% CI, 95% lower and upper confidence interval; COPD, Chronic Obstructive Pulmonary Disease.  Cox Proportional Hazard models fit with strata for gender, race, age, and ZIP code in SES-adjusted models, although for effect modification by SES, models adjusted for state-level SES but not ZIP code-level SES. Includes data for all Medicare beneficiaries between 2000-2008 living in the conterminous US. | | | |

# Figure S1. SES-adjusted exposure-response curves for 12-month moving average PM_2.5_ and specific causes of death

## (a) Cardiovascular-related diseases

The non-linear and linear association of PM_2.5_ on Cardiovascular-related mortality: All Cardiovascular, IHD, CBV and CHF. All analyses are conducted by Cox Proportional Hazard model with strata for age, gender, race, ZIP code and adjusted for SES. Bold solid line represents non-linear association estimated using restricted cubic spline (3 knots); dashed line represents 95% CIs for the non-linear, and dotted line represents linear association. RRs are based on comparisons to RRs for 0 $\mu g/m^{3}$_._ Abbreviations: IHD (Ischemic heart disease), CBV (Cerebrovascular disease), CHF (Congestive heart failure), SES (Socio-Economic Status), PM_2.5_ (particles with aerodynamic diameters < 2.5 $\mu m$).

## (b) Respiratory-related diseases

The non-linear and linear association of PM_2.5_ on Respiratory-related mortality: All Respiratory, COPD, and Pneumonia. All analyses are conducted by Cox Proportional Hazard model with strata for age, gender, race, ZIP code and adjusted for SES. Bold solid line represents non-linear association estimated using restricted cubic spline (3 knots); dashed line represents 95% CIs for the non-linear, and dotted line represents linear association. RRs are based on comparisons to RRs for 0 $\mu g/m^{3}$_._ Abbreviations: COPD (Chronic Obstructive Pulmonary disease), SES (Socio-Economic Status), PM_2.5_ (particles with aerodynamic diameters < 2.5 $\mu m$).

## (c) Cancer and Lung Cancer

The non-linear and linear association of PM2.5 on Cancer and Lung Cancer mortality. All analyses are conducted by Cox Proportional Hazard model with strata for age, gender, race, ZIP code and adjusted for SES. Bold solid line represents non-linear association estimated using restricted cubic spline (3 knots); dashed line represents 95% CIs for the non-linear, and dotted line represents linear association. RRs are based on comparisons to RRs for 0 $\boldsymbol{\mu g}/\boldsymbol{m}^{\mathbf{3}}$. Abbreviations: SES (Socio-Economic Status), PM2.5 (particles with aerodynamic diameters < 2.5 $\boldsymbol{\mu m}$).

# Figure S2. Sensitivity analyses: (a) SES-adjusted and (b) SES- and ozone-adjusted exposure-response curves for 12-month moving average PM_2.5_ and all-cause, CVD, respiratory and cancer deaths

## (a) SES-Adjusted models (no control for ozone)

## (b) SES- and ozone-adjusted models

For all cause of deaths, Cox Proportional Hazard model with strata for age, sex, race, ZIP code, and adjusted for area-level SES and warm season average of daily 1-hour maximum ozone exposures. Models fit for subset of Medicare beneficiaries (over 22 million) living near EPA ozone monitoring sites. Bold solid line represents non-linear association estimated using restricted cubic spline (3 knots); dashed line represents 95% CI for the non-linear. RRs based on comparisons to RRs for 0 $\mu g/m^{3}$_._

# Figu**re S3. SES-adjusted exposure-response curves for 12-month moving average PM_2.5_ and cause-specific mortality by effect modifier**

## (a) Sex

For each cause of death, we examined effect modification using interaction terms for gender (male, female) in the SES-adjusted models and with strata for age, race and ZIP code. Bold solid line represents non-linear association estimated using restricted cubic spline (3 knots); dashed line represents 95% CI for the non-linear, and dotted line represents linear association. RRs based on comparisons to RRs for 0 $\mu g/m^{3}$_._

## (b) Race

For each cause of death, we examined effect modification using interaction terms for race (white, black) in the SES-adjusted models and with strata for age, sex and ZIP code. Bold solid line represents non-linear association estimated using restricted cubic spline (3 knots); dashed line represents 95% CI for the non-linear, and dotted line represents linear association. RRs based on comparisons to RRs for 0 $\mu g/m^{3}$_._

## (c) Age

For each cause of death, we examined effect modification using interaction terms for age (>75, $\leq$75) in the SES-adjusted models and with strata for race, sex and ZIP code. Bold solid line represents non-linear association estimated using restricted cubic spline (3 knots); dashed line represents 95% CI for the non-linear, and dotted line represents linear association. RRs based on comparisons to RRs for 0 $\mu g/m^{3}$_._

## (d) Urbanicity

For each cause of death, we examined effect modification using interaction terms for urbanicity (urban, non-urban) in the SES-adjusted models and with strata for age, race, sex and ZIP code. Bold solid line represents non-linear association estimated using restricted cubic spline (3 knots); dashed line represents 95% CI for the non-linear, and dotted line represents linear association. RRs based on comparisons to RRs for 0 $\mu g/m^{3}$_._

## (e) SES

For each cause of death, we examined effect modification using interaction terms for income level (low-income, high-income) with strata for age, race, sex and ZIP code and adjusted for SES of state. Bold solid line represents non-linear association estimated using restricted cubic spline (3 knots); dashed line represents 95% CI for the non-linear, and dotted line represents linear association. RRs based on comparisons to RRs for 0 $\mu g/m^{3}$_._

# Figu**re S4. SES-adjusted exposure-response curves for 12-month moving average PM_2.5_ and all causes mortality with different number of knots**

For all cause of deaths, Cox Proportional Hazard model with strata for age, sex, race, ZIP code and adjusted for SES. Akaike information criterion (AIC) is also reported for the model fit: the lower, the better. Bold solid line represents non-linear association estimated using restricted cubic spline (3, 4 and 5 knots respectively); dashed line represents 95% CI for the non-linear. RRs based on comparisons to RRs for 0 $\mu g/m^{3}$_._

# Figu**re S5. SES-adjusted exposure-response curves for 12-month moving average PM_2.5_ and all causes mortality near monitoring sites.**

For all cause of deaths near monitoring sites, Cox Proportional Hazard model with strata for age, sex, race, ZIP code and adjusted for SES. Bold solid line represents non-linear association estimated using restricted cubic spline (3 knots); dashed line represents 95% CI for the non-linear. RRs based on comparisons to RRs for 0 $\mu g/m^{3}$_._

References

1. Cox DR. Regression models and life‐tables. J R Stat Soc Ser B. 1972;34(2):187–202.

2. Mittal S, Madigan D, Burd RS, Suchard MA. High-dimensional , massive sample-size Cox proportional hazards regression for survival analysis. 2014;207–21.

3. Liu DC, Nocedal J. On the limited memory BFGS method for large scale optimization. Math Program. 1989;45(1–3):503–28.

4. Andrew G, Gao J. Scalable training of L 1-regularized log-linear models. In: Proceedings of the 24th international conference on Machine learning. ACM; 2007. p. 33–40.

5. Croxford R. Restricted Cubic Spline Regression : A Brief Introduction. Toronto Inst Clin Eval Sci. 2016;1–5.

6. Harrell Jr FE, Harrell Jr MFE. Package 'Hmisc.' 2019.

7. Therneau TM, Lumley T. Package 'survival.' Surviv Anal Publ CRAN. 2014;
